# Role of emotional dispositions in modulating coping among artists

**DOI:** 10.1192/j.eurpsy.2026.11672

**Published:** 2026-07-31

**Authors:** H. Mezghani, N. Regaieg, F. Guermazi, S. Ajmi, I. Feki, J. Masmoudi, I. Baati

**Affiliations:** Psychiatry A Department, Hedi Chaker University Hospital, Sfax, Tunisia

## Abstract

**Introduction:**

Effective emotional regulation appears to be a significant challenge, particularly for students engaged in artistic training. However, affective traits interact with preferred coping mechanisms. Studying these interactions is essential to identify the underlying mechanisms and propose appropriate interventions.

**Objectives:**

The objective of this study is to investigate the impact of affective temperaments on the coping strategies used by artistic students.

**Methods:**

We conducted a cross-sectional, descriptive, and analytical study among students at the Institute of Arts and Crafts in Sfax (Tunisia), during the period from February 1^st^ to March 5^th^, 2025, based on an anonymous self-administered questionnaire containing sociodemographic, clinical, anda psychometric scale in its Arabic version: Tunisian TEMPERAMENT Scale (TEMPS) to measure affective temperaments.

**Results:**

The study included 60 students, 23 men and 37 women, with a sex ratio of 0.6. The median age of the sample was 20 years [19-22 years].

Using art as a means of channeling emotions and regular physical activity were significantly associated with hyperthymic temperament (p=0.03 and p=0.01, respectively).

Expressing emotions through art and seeking social support showed a statistically significant association with cyclothymic temperament (p=0.04 and p=0.045, respectively).

A significant association was found between depressive temperament and the tendency towards temporary isolation as well as the consumption of psychoactive substances (p=0.017 and p=0.01, respectively).

Anxious temperament was statistically associated with a marked tendency to avoid sources of stress as well as a propensity for rumination (p=0.01 and p=0.037, respectively).

**Conclusions:**

The influence of affective traits on the development of psychological support and stress management strategies appears significant. It therefore follows that these individual and emotional characteristics must be taken into account in order to support artistic students in their creative journey and their psychological well-being.

**Disclosure of Interest:**

None Declared

